# Design of a Multiplexed Analyte Biosensor using Digital Barcoded Particles and Impedance Spectroscopy

**DOI:** 10.1038/s41598-020-62894-z

**Published:** 2020-04-09

**Authors:** Shreya Prakash, Brandon K. Ashley, Patrick S. Doyle, Umer Hassan

**Affiliations:** 1 Department of Electrical and Computer Engineering, Rutgers, The State University of New Jersey, Piscataway, NJ 08854 USA; 2 Department of Biomedical Engineering, Rutgers, The State University of New Jersey, Piscataway, NJ 08854 USA; 3 Department of Chemical Engineering, Massachusetts Institute of Technology, Cambridge, MA 02139 USA; 4 Global Health Institute, Rutgers, The State University of New Jersey, Piscataway, NJ 08854 USA

**Keywords:** Electrical and electronic engineering, Biomedical engineering, Assay systems

## Abstract

Multiplexing allows quantifying multiple analytes in a single step, providing advantages over individual testing through shorter processing time, lower sample volume, and reduced cost per test. Currently, flow cytometry is the gold standard for biomedical multiplexing, but requires technical training, extensive data processing, and expensive operational and capital costs. To solve this challenge, we designed digital barcoded particles and a microfluidic architecture for multiplexed analyte quantification. In this work, we simulate and model non-fluorescence-based microfluidic impedance detection with a single excitation and detection scheme using barcoded polymer microparticles. Our barcoded particles can be designed with specific coding regions and generate numerous distinct patterns enabling digital barcoding. We found that signals based on adhered microsphere position and relative orientation were evaluated and separated based on their associated electrical signatures and had a 7 µm microsphere limit of detection. Our proposed microfluidic system can enumerate micron-sized spheres in a single assay using barcoded particles of various configurations. As representation of blood cells, the microsphere concentrations may provide useful information on disease onset and progression. Such sensors may be used for diagnostic and management of common critical care diseases like sepsis, acute kidney injury, urinary tract infections, and HIV/AIDS.

## Introduction

Whole blood samples provide imperative data useful for healthy monitoring and understanding disease progression for individuals in critical care settings. Each cell type within the blood has unique properties and methods of isolation, including their relative concentration. Currently, as disease onset occurs, a Complete Blood Cell count (CBC) is often the first step for evaluating a patient’s status^1^. However, the CBC denies the whole story, as different cellular behaviors, organelle or membrane properties, and mechanical responses by the cells are significantly better indicators for accurate disease determination^2,3^. More specialized, multiplexed techniques targeting these blood cell biomarkers may be the key for robust and expedient diagnosis.

Most diagnostic equipment requires large blood volumes to measure each targeted analyte separately. In these cases, properties of the blood biomarkers may change and will not satisfy test requirements, burdening both patients of compromised function and healthcare providers conducting the blood extractions^4^. While gathering information on more analytes provides more accurate diagnostics, usually experiment complexity and data analysis increases alongside it. As a solution. a multiplexing quantification approach for proteins, nucleic acid sequences, or cytokines overcomes such issues by detecting each biomarker with the same source and sample volume. These abilities enable multiplexing to obtain high density information in minimal time along with low sample volume and less cost^5^. To increase multiplexing solutions for biomedical diagnostics, however, they must transition to low-cost, easily manufactured, and deliverable devices.

Currently, flow cytometry is employed for obtaining heterogenous cell sample characteristics beyond a CBC as a mainstream multiplexing system. It uses optical methods for adjudicating multiple physical and chemical cell properties in a fluid stream through different visible light detectors^6^. Flow cytometry is widely used for immunophenotyping many clinical samples like whole blood^7–9^, bone marrow^10^, cerebrospinal fluid^11^, urine^12^ and hematological malignancies^13^. Although flow cytometry is ubiquitously used as the diagnosing standard, it has severe shortcomings in cost, unable to quantify secreted or dissolved compounds, complicated data analysis, and poor receiver operating curves for in-the-field use^2,7,14–18^. Several researchers are pioneering miniaturized, novel methods as point-of-care alternatives to flow cytometry, including fluorescent microfluidics for nucleic acid and protein quantification^19–21^, droplet-based microfluidics for secreted biomarker analysis^17,22,23^, planar microarrays with micro-engraving techniques for temporal cell behavior evaluation^24–27^, and barcoded microchip devices for membrane and cytosolic protein detection^28–30^. Such proceedings can be directed for treating complicated and changing diseases requiring multiple biomarker determinations like sepsis^5,7,15,31,32^, HIV/AIDS^9,33–35^, acute kidney injury^36–38^, urinary tract infections^12,22^, and malignant tumors^26,30,39–41^. While these technologies may provide adequate capabilities in future developments, many are still lacking in adaptive biomarker targeting, low-cost manufacturing, or reducing blood sample volumes required for testing^7,17,18,42^.

Therefore, it is necessary to develop a low-cost, compact, portable device for multiplexing analytes of various size and property. Here, we model and simulate polydimethylsiloxane (PDMS)-based barcoded particles fabricated using stop-flow lithography^43,44^ for biomarker detection. Barcodes for specific PDMS particles can correspond with unique functionalization protocols directed towards individual biomarkers, allowing extrapolation on their identification, size, quantity, and binding orientation. Barcode regions and analytes themselves can be identified using impedance-based detection, providing advantages over fluorescent, quantum dot, and surface-enhanced plasmon resonance techniques through less expensive protocols, single excitation/detection sources, and having superior multiplexing capabilities^14,23,27,45–47^. Moreover, a microfluidic scheme requires less sample volume and is miniaturized using microfabrication techniques, allowing simple manufacturing and therefore expediated transport to more in-need populations. In this work, we evaluate the design and simulate impedance-based microfluidic behaviors with novel barcoded particles for cell surface receptor detection.

## Results

### Simulating microsphere impedance detection through the microfluidic channel

**Figure 1:**
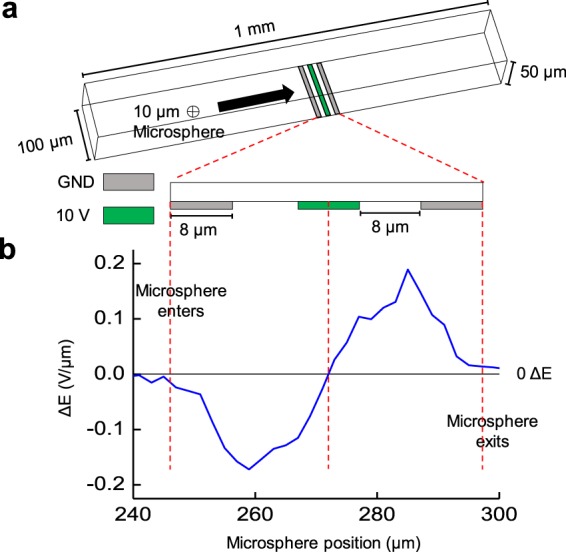
Overview of microfluidic impedance detection system. (**a**) Three-dimensional view of the microfluidic impedance detection channel with three coplanar electrodes. Here, the length, width, and height of the channel are 1 mm, 100 µm, and 50 µm respectively, while each platinum electrode is 8 µm in width and spaced 8 µm apart (scheme not to scale). The outer platinum electrodes are grounded detectors (GND, silver) while the interior electrode supplies a 10 V AC input (green). **(b)** The differential bipolar electrical signature, ΔE, of a single 10 µm glass microsphere flowing left to right across the electrodes in the microfluidic channel. ΔE is obtained by taking the difference between recorded voltage between the first GND to 10 V input and the 10 V input to the second GND. Here, the peaks for ΔE are directly proportional to microsphere size.

Figure 1a shows a 3-D view of the microfluidic impedance detection environment, with simulation results modeling a cell passing through the channel. Here, the central platinum electrode (green) is excited by a 10 V AC signal and the impedance is detected between the exterior platinum electrode pair (silver). As the modeled, inert microsphere crosses over the electrode area, a differential bipolar pulse directly related to the impedance is generated (Fig. 1b), which is indicative for heterogeneous cell population characterization based on cellular diameters.

The detection system consists of three coplanar platinum electrodes, with a width and spacing between electrodes of 8 μm (Fig. 1a). Additionally, the microfluidic channel has a 50 μm height, 100 μm width, and 1 mm length, while an excitation voltage of 10 V AC is provided to the central electrode and the impedance measurement is conducted between the peripheral electrodes. As the microsphere crosses over the coplanar electrodes, the electric field lines are disrupted, increasing the impedance which is the grounded electrode’s output in the collected impedance shift. Using two detecting electrodes allows for a differential, bipolar pulse which can be normalized to reduce intrinsic noise. To test the proposed design, a microsphere 10 μm in diameter travels through the microfluidic architecture simulated in COMSOL Multiphysics to generate the electrical signature for the blood cell. As represented by the bipolar pulse generated (Fig. 1b), the microfluidic scaffold is sensitive enough to detect a single cell passing through the channel.

**Figure 2:**
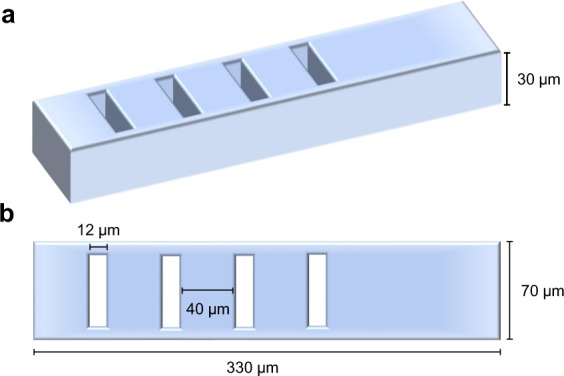
Schemes of fabricated barcoded particle. (**a**) 3-Dimensional representation of the barcoded particle modeled using polydimethylsiloxane (PDMS) material, with a depth of 30 µm. **(b)** Top-down (transverse) view of the barcoded particle with defined geometries of 70 µm in width and 330 µm in length, along with “barcoded” regions which are the absence of PDMS that are 12 µm in width and are evenly spaced 40 µm apart. The barcodes are aligned asymmetrically with the particle to produce a non-mirrored electrical response for the upcoming experiments.

Following the microsphere detection, we now model a proposed novel design for an asymmetric barcoded particle with four coding regions, each generating distinct electrical signatures. As a model, the presence and absence of coding regions produces distinguishable bipolar pulses, hence ensuring differential detection through various barcode sequence combinations. Figure 2 illustrates a 3-D (Fig. 2a) and top-down (Fig. 2b) view of the barcoded particle with four coding regions. Barcoded particle dimensions are 330 µm in length, 30 µm in height, and 70 µm in width. Furthermore, the removed barcoded regions are 12 µm in width and are spaced 40 µm apart. (Fig. 2). As referenced earlier, physical barcoded particles are fabricated via stop flow lithography^43^. In this method, the particles arise from a stationary monomer PDMS film using conventional photolithography, and barcoding regions are displaced in the PDMS via a micron-sized high-pressure air nozzle.

### Characterizing the barcoded particle absent of adhered materials

**Figure 3:**
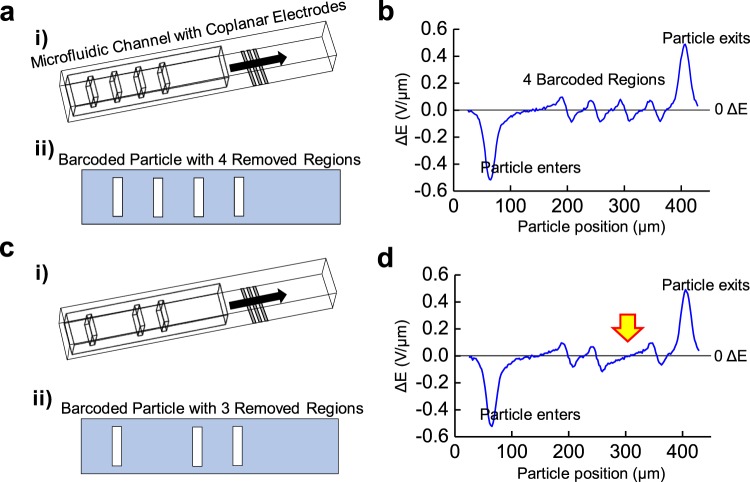
Barcoding changes ΔE response relative to position on the particle. (**a**) (**i**) Mesh view of the microfluidic channel with the 4-barcoded particle modeled through COMSOL. Here, the particle passes over outer output platinum electrodes, followed by a middle voltage input platinum electrode (all in silver), with the electrode widths of 8 µm and spaced 8 µm apart. **(a)** (**ii**) Transverse view of the barcoded particle with 4 coding regions. **(b)** The differential bipolar electrical signature, ΔE, measured as the barcoded particle with 4 coding regions passes across the electrode region. Here, the largest peaks correspond to the particle entering and exiting the electrode region, while the 4 minor peaks represent barcode detection. **(c)** (**i**) Mesh view of the microfluidic architecture with 3-barcoded particle, removing one of the middle barcoded region. **(c)** (**ii**) Transverse view of barcoded particle with 3 coding regions. **(d)** The differential bipolar electrical signature, ΔE, measured as the barcoded particle with 3 coding regions passes across the electrode region. Here, the lack of middle barcode is revealed, as indicated by the yellow arrow, and the two barcoded particle identities can be differentiated.

After establishing the microfluidic architecture and generalized form for our proposed barcoded particles, designs with four and three coding regions are simulated in COMSOL Multiphysics to determine their unique bipolar electrical signatures (Fig. 3). Here, the rectangular asymmetric barcoded particles move through the microfluidic channel with three platinum electrodes located below the channel. While flowing through the microfluidic architecture, each barcoded particle generates a distinct electrical signature and comprises of a distinct entrance and exit pulse, corresponding with the certain number of minor peaks as a function of particle position across the electrode center (Fig. 3b,d). Specifically, barcode particle signatures entering and exiting the electric field, as well as barcode regions on the particle, output 0.564 V/µm and 0.098 V/µm differential pulses, respectively.

As an example, the barcoded particle with four coding regions (defined with the barcode sequence 1,1,1,1) reveals four minor peaks in the electrical signature (Fig. 3b), while the barcoded particle with three coding regions (with a barcode sequence of 1,0,1,1) has three minor peaks (Fig. 3d). The particle lacking a barcode region in the 2′ position has a correspondingly absent electrical peak indicated by the yellow arrow. Detecting the bipolar electrical pulse measured as the barcoded particle moves across the electric field allows distinct and identifiable peaks which proportionally relate to the number and spacing of barcoded regions.

### Sensitivity evaluation for a single microsphere conjugation

**Figure 4:**
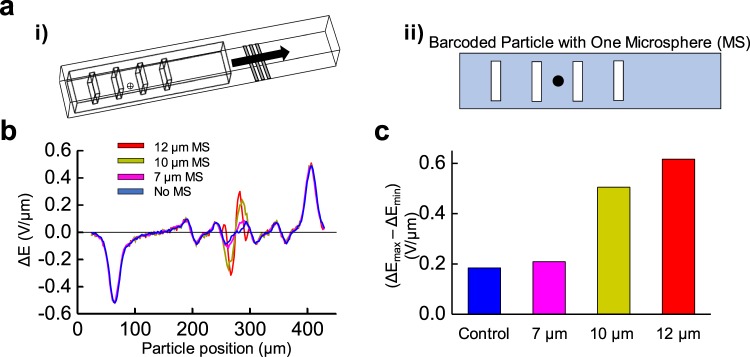
Barcoded particle recognition using microspheres of varying sizes. **(a) (i)** Mesh view of the microfluidic channel consisting of the barcoded particle with one microsphere (MS) attached on the bottom of the barcoded particle with the platinum coplanar electrodes (silver). **(a) (ii)** Transverse view of the barcoded particle with one adsorbed MS centered between the two center barcode regions. **(b)** COMSOL modeling of the differential bipolar electrical signature, ΔE, measured for the barcoded particle with no adsorbed MS (blue), one 7 µm adsorbed MS (pink), one 10 µm adsorbed MS (dark yellow), and one 12 µm adsorbed MS (red) across the length of the barcoded particle. **(c)** Bar graph representing the difference between the largest positive and negative electrical peaks for each sized microsphere. Here, MS size is linearly correlated with greater electrical signature recorded (R-squared = 0.98).

A single microsphere (MS) which is modeled after a blood cell with inert electrical properties is conjugated on the center of the barcoded particle’s bottom face. At this position, our study analyzed MS with 7, 10, and 12 μm diameters (Fig. 4a). Simulated in COMSOL Multiphysics, the test determines the sensitivity and limit of detection for the barcoded particle when a single MS is conjugated of varying size. Figure 4 illustrates MS conjugation to the barcoded particle and their respective electrical signatures.

It is observed that a 12 μm MS produces a change in the electrical signature 3.5 times the original electrical signature of the minor peaks from the barcoding regions (i.e., no MS conjugated). Similarly, a 10 μm MS conjugated results in 2.8 times increase in electrical signature peak, while the 7 μm MS only saw 1.4 times increase. By evaluating the difference from maximum bipolar peak signatures from the MS relative to their minimum peaks (∆E_max_ − ∆E_min_, Fig. 4c), a linear relationship can be obtained to correlate with the MS size: (1), with R_MS_ representing MS radius

1 $$(\Delta {E}_{max}-\Delta {E}_{min})=0.1658{R}_{MS}-0.3578$$

By setting the ∆E_max_ − ∆E_min_ equal to the signature from the barcoding region (0.17 V/μm), and setting a threshold value for detection of 1.1 times greater, the barcoded particles’ current MS limit of detection is 6.5 μm. The peaks indicating the particle entering and exiting the electric field area are neglected for ∆E_max_ − ∆E_min_ determination.

### Orientation analysis for conjugated microspheres

**Figure 5:**
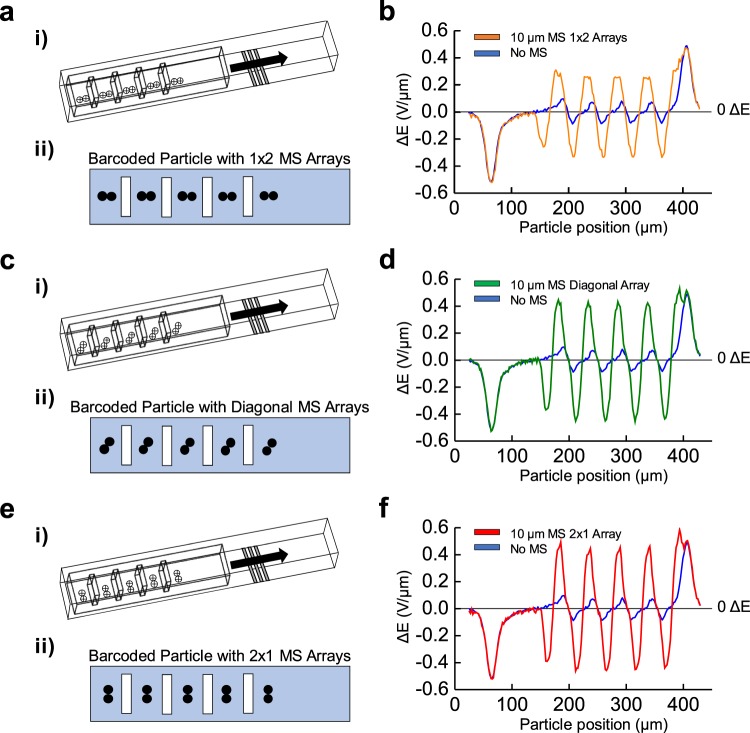
Barcoded particles revealed different ΔE responses for microsphere arrays of different arrangements. **(i)** Mesh view of the microfluidic channel with the barcoded particle and an array of microspheres (MS); in **(a)** rows of two across, **(c)** with a diagonal array of two MS, and **(e)** in columns of two MS attached to the bottom of the barcoded particle. **(ii)** Transverse view of the barcoded particle with an array of MS; in **(a)** rows of two between each barcoded region, **(c)** in columns of two MS, and **(e)** with a diagonal array of two MS. COMSOL modeling of the differential bipolar electrical signature, ΔE, measured for the barcoded particle with no adsorbed MS (blue), **(b)** MS arrays two across (orange), **(d)** with diagonal arrays of two MS (green), and **(f)** MS arrays of two in columns (green). Here, a 10 µm MS was modeled, and the different orientations of MS assembled on the barcoded particle can be visualized through unique electrical responses.

The orientation of the conjugated microspheres at the bottom of the barcoded particle is further studied to determine changes in electrical signature peaks. A MS array is conjugated to the bottom face of the barcoded particle in different possible orientations for the following simulation through COMSOL Multiphysics (Fig. 5a,c,e). Specifically, the arrangements are MS 10 μm in diameter with arrays of two aligned axially (1 × 2 MS array, Fig. 5a), aligned diagonally (diagonal MS array, Fig. 5c), and aligned orthogonally with barcoded particle length (2 × 1 MS array, Fig. 5e) on the bottom face of the barcoded particle.

It is observed that the electrical signature of the conjugated 1 × 2 MS array is 3.8 times the original signature without any MS attachment (Fig. 5b). Similarly, the diagonal and 2 × 1 MS microarrays saw 5.5 and 5.8 times the original signature, respectively (Fig. 5d,f). While the change between the diagonal and 2 × 1 MS array is small, their margins of error allow the patterns to be significantly distinguishable. Another revelation is MS arrays closer or completely aligned with the electrodes and orthogonal to flow direction (i.e., the 2 × 1 MS microarrays) displayed the largest peak electrical signatures relative to other orientations, as greater MS surface area by the array is placed at the peak electrical position.

**Figure 6:**
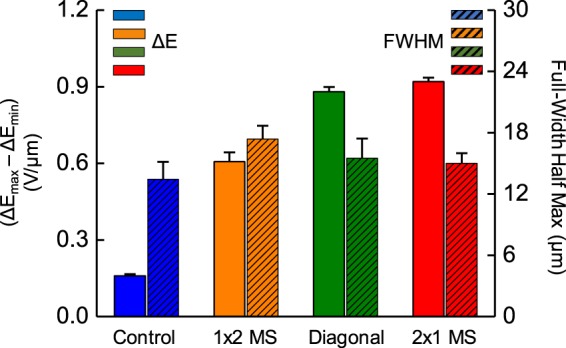
Observing correlations between peak ΔE and full-width half maximum (FWHM) with MS orientation. Bar graph of maximum differences between positive and negative peaks (solid colors) as well as the average FWHM distances of those peaks (diagonal patterns) corresponding to 10 µm sized microsphere (MS) arrays in 1 × 2 arrays (orange), diagonal arrays (green), and 2 × 1 arrays (red).

Another metric, the full-width half max (FWHM), defines the spread of MS peaks across the distance the particle travels and can further characterize both MS size and position (Fig. 6). For the 1 × 2, diagonal, and 2 × 1 MS arrays, there was a FWHM of 17 μm, 15.5 μm, and 15 μm respectively (Fig. 6). The 1 × 2 MS array had the largest FWHM, as the longer distance with MS arrays aligned with the barcoded particle length over the electric field area will correspondingly spread the detection signal across longer spatial positions. Hence, it can be observed that orientation of the conjugated MS arrays influences the associated electrical signature, both in peak value and peak spread. To modulate the ability of MS conjugation on the barcoded particle with different orientations, changing the area between barcode regions and therefore space for possible MS conjugation can affect the probability of 1 × 2 versus 2 × 1 MS arrays.

### Using top-bottom electrode configurations for multi-face microsphere attachment

Up to this point, all simulations were evaluating electrical pulses for MS conjugation on the bottom face of the barcoded particle, corresponding with the platinum electrode placement on the channel floor. With this configuration, MS arrays of 10 μm in diameter attached on the top face of the barcoded particle did not significantly influence electrical pulse peak or spread (Supplemental Fig. 1). Such conditions would negatively impact the impedance-based detection strategy as the PDMS particle insulates the MS from electrode detection, while also placing the MS farther away from the electrodes in a weaker electric field region.

**Figure 7:**
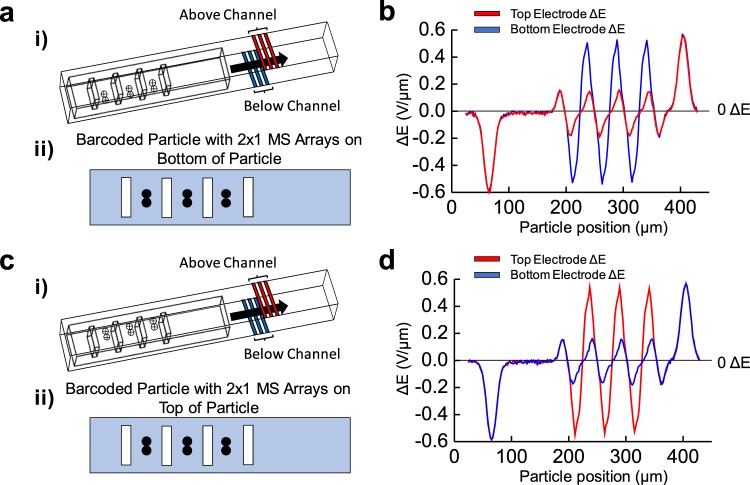
Using planar electrodes above and below the microchannel. (**i**) Mesh view of the microfluidic channel now with electrodes both below (highlighted in blue) and above (highlighted in red) the channel and the barcoded particle with an array of 2 × 1 10 µm microspheres (MS) attached **(a)** on the bottom and **(c)** top of the barcoded particle. **(ii)** Transverse view of the barcoded particle with 2 × 1 MS arrays attached to **(a)** the bottom and **(c)** top of the particle. COMSOL modeling of the differential bipolar electrical signature, ΔE, measured for the barcoded particle conjugated with the 2 × 1 MS array at **(b)** the bottom and **(d)** top of the barcoded particle from the bottom electrode (blue) and the top electrode (red). With a top and bottom electrode configuration, MS attachment can be detected regardless of transverse face attachment to the barcoded particle.

As a solution, a novel top-bottom sensing architecture shown by Fig. 7 will be modeled, discerning MS presence regardless of their conjugation face. Here, the microfluidic channel comprises of microfabricated coplanar platinum electrodes on both the top and bottom of the channel (Fig. 7a,c). For each set, the middle electrodes will input a 10 AC V signal, while the impedance measurement will be carried between the pair of peripheral grounded electrodes. Figure 7 illustrates the barcoded particle conjugated with the microsphere array in the top-bottom electrode configuration, with the top electrodes and data highlighted in red and bottom electrodes and data highlighted in blue. Top electrodes will be fabricated by micropatterning platinum directly above the PDMS channel, and extended metal pads patterned on the glass substrate which supports the PDMS will facilitate circuit connection to such electrodes. Since the top and bottom electrode formations will work independently, vertical alignment with respects to each other will not be a requirement.

Using 2 × 1 MS arrays attached to the bottom (Fig. 7a,b) and top (Fig. 7c,d) faces of the barcoded particle, the different planar electrode groups may be evaluated for their MS detection ability. For MS arrays on the bottom face, there is a significantly larger electrical response detecting them from the bottom electrode (Fig. 7b, in blue) versus the top electrode (Fig. 7b, in red). A similar trend follows MS arrays on the top face; a significantly greater electrical response from the top electrode compared with the bottom (Fig. 7d). Interestingly, the average electrical peaks are nearly identical for the electrodes detecting MS arrays attached on the complementary face of the barcoded particle (i.e., the electrical signal from the MS arrays attached to the bottom face detected by the bottom electrode were equivalent to signal from the MS arrays attached to the top face detected by the top electrodes). Such a configuration utilizing dual-electrode groups, along with correlating electrical field peaks and spreads to MS size and position, grant insight and knowledge into expected signatures with *in vitro* diagnostics and sensing with the PDMS-based barcoded particles of attached cells.

## Discussion

A non-fluorescent microfluidic architecture with a single excitation and detection scheme using novel barcoded particles is proposed in this study. The designed barcoded particle produces distinct electrical signatures when they travel through a microfluidic impedance detection system. With four coding regions, the barcoded particle is suited for multiplexing capabilities, with code variabilities allowing for over 9 distinguishable particles. Furthermore, the asymmetric nature of the particle significantly increases multiplexing capabilities to 15 unique variants, as barcodes of unequal spacing between particle edges allow for barcode combinations dependent on the reading frame direction. This reading frame can be differentiated, though, from the spacing by which the barcode sequences begin after peaks from the particle entering the electric field, and can be represented by Eq. 2:

2 $$N={\sum }_{k=1}^{n}{}_{k}{}^{n}C$$

where N is the distinct number of combinations, n is the number of barcoded regions, and k represents the number of possibilities (i.e., either a barcode is present or not). Future designs may propose more barcode regions, which could exponentially increase the number of distinct particles. Even an asymmetric particle with 7 barcoded regions may have 127 different combinations, permitting evaluation of that many biomarkers from a single apparatus and detection system. While the need for identifying over 100 biomarkers in a sample is unnecessary for many diseases, it may prove useful for sepsis which has related over 170 biomarkers to potential diagnosing capabilities^48^.

From our results, the proposed microfluidic architecture has a 6.5 µm limit of detection for microsphere conjugation to the barcoded particle. Such size is equivalent or smaller than individual immune cells found in blood, and can be effective for quantifying multiple cell types across different barcoded particles. Microsphere placement orientation effects were also investigated, and showed different but predictable peak magnitudes and distributions. Finally, by employing a top-bottom microelectrode apparatus, microspheres are detectable both above and below the barcoded particle. While the fabrication of top and bottom electrodes for microfluidic applications are infrequent, recent studies have found effective means to produce results using glass-embedded electrodes sandwiching PDMS-based channels, and we believe such methods are possible for *in vitro* design experiments^49^. Furthermore, our top-bottom electrode configuration is independent of vertical alignment between electrodes as both systems emit/detect their own electric field areas, reducing the burden of micron-scale alignment protocols and promoting simpler microfluidic manufacturing.

Building on our design simplicity, we emphasize our detection system having multiplexing capabilities through one sensing scheme and one target represented with the barcoded particle. Other multiplexing mechanisms require several physical controls which must be maintained and accounted for to perform accurate detection, such as proper filters or dyes corresponding with predetermined biomarkers in fluorescence-based systems^14,15,19–21^ and unique and time consuming fabrication protocols for other impedance-based classifications^29,50^. Contrastingly, the only variation for our barcoded particle mechanism is the barcode sequence produced during stop-flow lithography, which can be easily modulated and allow for rapid production of defined particles^43^. Other directions to improve multiplexing include parallelization of the detection systems, such as barcoding of the microfluidic structures themselves^29,51,52^. While accurate for multiple biomarker identification on-chip, their intrinsic designs lead to larger structure area, require larger sample volumes, and electrically-based parallel schemes result in increasing overall electrical resistance across the chip and dampen output signal^45,47^. Most critically may be the sample volume, which must be optimized to a minimum for many point-of-care settings, where blood collection is significantly taxing. However, our design requires equivalent sample volume analyzing one biomarker versus 100 biomarkers, and our one detecting scheme for every barcoded particle configuration does not affect or adversely increase detecting area. Indeed, the only variation for our multiplexing strategy is through the barcode sequence fabrication, allowing for modest quality control and the proper scope for simple, easily produced yet accurate diagnostic devices.

For future directions and strategies, we aim to functionalize physical barcoded particles with specific antibodies critical to cell-surface biomarker attachment. Our group is directly extending upon the results from this study to characterize and study multiplexing capabilities for point-of-care sepsis diagnosis. Specifically, we intend to correlate different barcode configurations to unique antibodies which target biomarkers related to sepsis expressed on immune cell membranes, including CD11b, CD66, and more. A PDMS-based microfluidic apparatus using the same dimensions from this paper will be fabricated to both isolate immune cells from complete blood and measure their electrical responses after attaching to functionalized barcoded particles using the proposed impedance detection system. From here, a relationship can be identified with multiple biomarkers simultaneously using different barcodes and the degree of cell attachment to the particles, and controls such as flow cytometry and enzyme-linked immunosorbent assays (ELISA) will determine the rigor and accuracy of our system. Additionally, while the results were not exhaustive in microsphere attachment configurations, efforts are continuing to develop a multifeatured selection algorithm to readily determine their orientation based on electric peak magnitude and peak spread. Following functionalization techniques, we intend to perform *in vitro* studies for different cell types and explore magnetization effects conjugated microspheres have as they pass over the coplanar electrodes in the channel. With improved detection sensitivity, we aim to quantify different proteins expressed by the cell surface using the distinct signatures of the barcoded particle. Our group’s barcoded particle approach for multiplexing has a promising future in multiple point-of-care, low-cost applications and can be a solution for currently inadequate disease diagnosis standards.

## Methods

### Microfluidic channel modeling

Channel dimensions were 100 µm in width, 50 µm in height, and 1000 µm in length. Through COMSOL Multiphysics software (Burlington, MA), water was selected to immerse the defined channel volume. Water was selected for it’s predetermined and well-defined properties in COMSOL software, and additional studies reveal it behaved similarly to fluids conventionally used in biological microfluidics such as phosphate-buffered saline (PBS) (Supplemental Information Fig. 3). Specific water material properties have an 80 relative permittivity (ε_r_), 0.89 mPa*s dynamic viscosity (µ), 5.5*10^−6^ S/m electrical conductivity (σ), 4.186*10^3^ J/(kg*K) heat capacity at constant pressure (C_p_), 997 kg/m^3^ density (ρ), and 0.598 W/(m*K) thermal conductivity at 25 °C (k).

Positioned on the channel floor 380 µm from the start of the channel are three structures 8 µm wide, 50 µm long, and 0.1 µm in height representing the platinum electrodes. Material properties include a 7 relative permittivity (ε_r_), 8.9*10^6^ S/m electrical conductivity, 8.8*10^−6^ K^−1^ coefficient for thermal expansion (α), 133 J/(kg*K) heat capacity at constant pressure (C_p_), 2.15*10^4^ kg/m^3^ density (ρ), 71.6 W/(m*K) thermal conductivity at 25 °C (k), 1.68*10^2^ MPa elastic modulus (E), and 0.38 Poisson’s ratio (ν). Under the Electrostatics module in COMSOL, water filling the microchannel geometry was defined with zero charge and supplied with an initial value of 0 V. Charge was conserved through the constitutive relationship between electric field strength and relative permittivity:

3 $$E=\frac{D}{{\varepsilon }_{0}{\varepsilon }_{r}}$$

where E is the electric field, D is dielectric constant for water, ε_r_ is relative permittivity for water, and ε_0_ is the relative permittivity of free space. Electric field taken from one outer electrode to the center applied electrode (24 µm) further defines the measured voltage potential (∆V):

4 $$\Delta V=Ed$$

However, as the electric field varies in channel height, a non-linear behavior is taken on and is visualized by Supplemental Fig. 2. Such electric field variations may influence signal properties for particles flowing through the channel at different but predictable heights and was explored in the Supplemental information (SI Fig. 3).

Further through the Electrostatics module, outer electrodes (Fig. 1a) were grounded, i.e., not supplied voltage, while the center electrode was supplied a 10 V potential. Through the COMSOL Mesh module, an extra fine element size was selected under a Physics-controlled mesh, which provided thorough detail in output data while mitigating laborious and time-consuming analysis through more specific element sizes.

### Barcoded particle modeling

Barcoded particle dimensions modeled in COMSOL Multiphysics are 330 µm in length, 30 µm in height, and 70 µm in width. The removed barcoded regions are 12 µm in width, 50 µm in both depth and length and are spaced 40 µm apart. (Fig. 2). Physical PDMS-based barcoded particles are fabricated by stop-flow lithography and will travel through the center of the microfluidic channel aligned axially with channel length based on laminar, pressure driven flow generating the fastest fluid velocity at the channel’s center. For simulations, the PDMS barcodes have material properties of 2.75 relative permittivity (ε_r_), zero electrical conductivity, 9*10^−4^ K^−1^ coefficient for thermal expansion (α), 1460 J/(kg*K) heat capacity at constant pressure (C_p_), 9.7*10^2^ kg/m^3^ density (ρ), 0.16 W/(m*K) thermal conductivity at 25 °C (k), 0.75 MPa elastic modulus (E), and 0.49 Poisson’s ratio (ν). Under the Study module in COMSOL, a parametric sweep was conducted as the x-position of the particle in unison with any attached microspheres or array of microspheres varied from 25 µm to 429 µm in x and recorded every 2 µm. The output solution was electric potential across the globally-defined geometric parameters, while the input voltage was defined with a 300 kHz frequency over the study period. Convergence with the study was resolved after 4 iterations, and electric potential data was evaluated and exported from the Results module following study analysis.

### Microsphere modeling

Microsphere dimensions were defined solely by particle radius through COMSOL Multiphysics. For results from Fig. 1, microsphere radius was set as 5 µm, while microsphere radius was 3.5, 5, and 6 µm for the 7, 10, and 12 µm microspheres used from Fig. 4. Further studies in orientation analysis and using top-bottom electrode configurations incorporated microspheres with 5 µm radii. For microsphere material, glass was selected. Using a 300 kHz voltage frequency, the electric signal is unable to penetrate through a cell’s phospholipid bilayer, which under these conditions behaves as electrically inert. With this assumption, a glass material with similar size to a cell reduces the strain of modeling the 20 nm cell membrane and would hamper simulation progress, performed similarly to others in the field^53^. All material parameters remained constant for microsphere modeling, including a 4.2 relative permittivity (ε_r_), 1*10^−14^ S/m electrical conductivity, 9*10^−4^ K^−1^ coefficient for thermal expansion (α), 730 J/(kg*K) heat capacity at constant pressure (C_p_), 2.21*10^3^ kg/m^3^ density (ρ), 1.4 W/(m*K) thermal conductivity at 25 °C (k), 70 GPa elastic modulus (E), and 0.21 Poisson’s ratio (ν), and 1 relative permeability (µ).

## Supplemental Information

Supplemental Information.
