# Supplementary material for: Design of a Multiplexed Analyte Biosensor using Digital Barcoded Particles and Impedance Spectroscopy
Source: Sci Rep. 2020 Apr 9;10:6109. doi: 10.1038/s41598-020-62894-z (PMC7145859; doi:10.1038/s41598-020-62894-z)
Supplement: Supplementary file 1 — Supplemental Information. [file 41598_2020_62894_MOESM1_ESM.pdf]

Design of a Multiplexed Analyte Biosensor using Digital Barcoded Particles and Impedance Spectroscopy

†Shreya Prakash[1], †Brandon K. Ashley[2], Patrick S. Doyle[3], Umer Hassan[1,2,4*]

[1]Department of Electrical Engineering, Rutgers, State University of New Jersey, Piscataway, NJ, 08854, USA

[2]Department of Biomedical Engineering, Rutgers, State University of New Jersey, Piscataway, NJ, 08854, USA

[3]Department of Chemical Engineering, Massachusetts Institute of Technology, Cambridge, MA, 02139, USA

[4]Global Health Institute, Rutgers, State University of New Jersey. Piscataway, NJ, 08854, USA

*Corresponding Author: umer.hassan@rutgers.edu; Tel (848) 445-2164

†These authors have equally contributed

Supplemental Information

Microsphere conjugation to the top surface of the barcoded particle

The microsphere array is conjugated at the top surface of the barcoded particle to check the sensitivity of the microfluidic detection system. For this study, two microspheres 10 μm in diameter are aligned as a 2x1 array. The barcoded particle with the conjugated microspheres travels through the microfluidic architecture to obtain the electrical signature. However, the electrical signature of the barcoded particle did not reflect significant change compared to no microspheres are conjugated to the barcoded particle. The detection system (micro fabricated coplanar electrodes) were unable to detect the microspheres attached at the top surface of the barcoded particle as shown in Supplemental Figure 1. The detection system is present at the bottom surface of the microfluidic channel and hence is insensitive to the microspheres conjugated to the top of the barcoded particle. Therefore, it is imperative to design the microfluidic architecture that could detect the presence of microspheres conjugated either on the top or bottom surface of the barcoded particle.

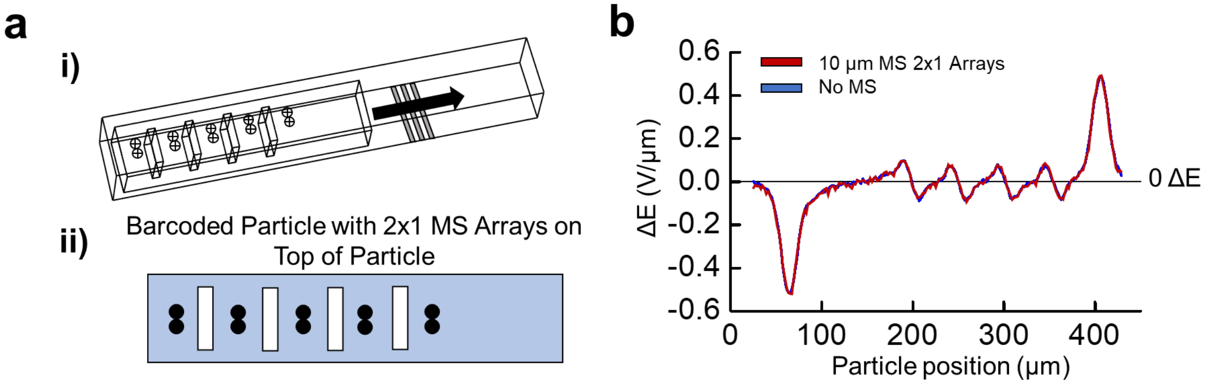

SI Figure 1: Microspheres attached on top of the barcoded particle using bottom-positioned electrodes alone. (a) (i) Mesh view of the microfluidic channel with the barcoded particle and an array of 2x1 10 μm microspheres (MS) attached on the top of the barcoded particle. **(a) (ii)** Transverse view of the barcoded particle with an array of MS attached to the top of the barcoded particle. **(b)** COMSOL modeling of the differential bipolar electrical signature, ΔE, measured for the barcoded particle conjugated with the 2x1 microsphere array at the top surface. Here, the electrodes are unable to detect the MS.

Electric field visualization using COMSOL Multiphysics

SI Figure 2 shows an arrow plot of voltage values based on electric potential from the central platinum voltage input (a), as well as an electric field distribution (b) generated using COMSOL Multiphysics software. For both, it is realized that lower values expose the channel at higher heights, which electric field values significantly lower even microns in height above the electrodes. This is attributed to the low electrical conductivity of water as the fluid volume in the channel ($5.5*10^{-6}$ S/m), which reduces voltage propagation. As the breakdown electric field value for a cell membrane is $4.0*10^{6}$ V/m, the electric field for the proposed system fall far lower, with an average in-channel value of 176 V/m[1]. This maintains cell viability from electrical and likewise thermal effects after passing through the channel and exposure to the voltage-generating electrode, as electrical and thermal properties are directly related to the electrical conductivity for water.

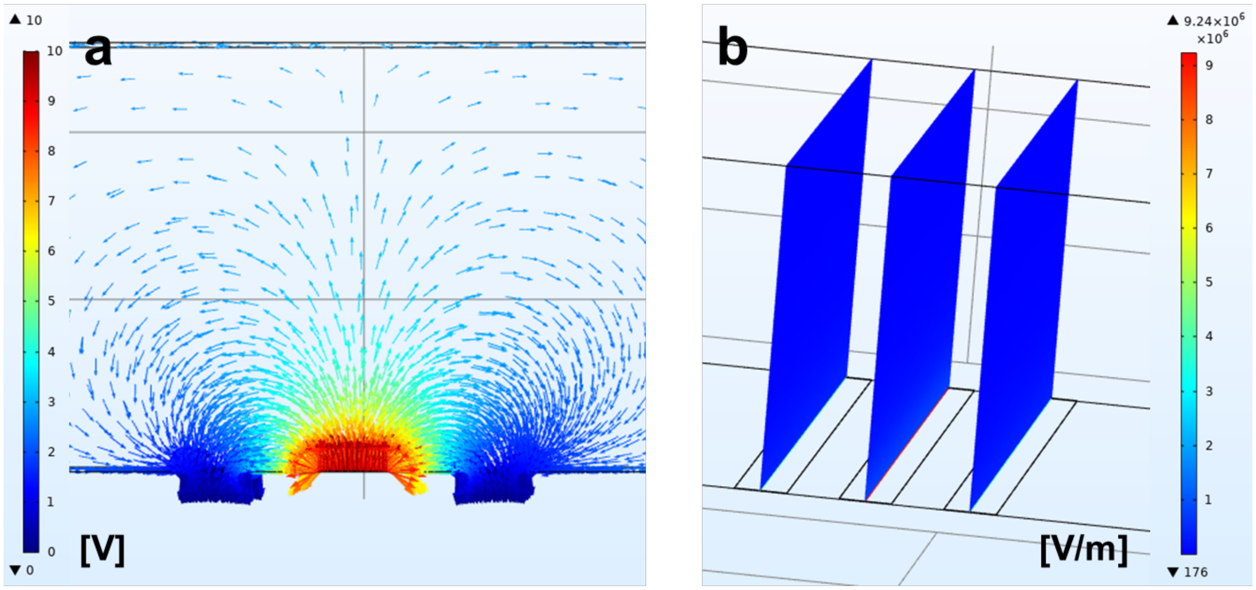

SI Figure 2: Electric field line representation modeled through COMSOL color map. (a) Voltage values (in volts) around electrodes from 0 to 10 V in COMSOL with a X-Y plane view, where a non-linear distribution is realized (b) Electric field model (in volts per meter) in the Y-Z plane above the electrodes in COMSOL. Here, electric field strength is strongest directly on the electrodes, but weakens rapidly up the channel.

Electrical signature characterizations through varying simulation parameters

As electric field strength varies nonlinearly with respect to channel height in our microfluidic model, it is expected that variations in barcode height may significantly alter signal peak range and spread. Here, we model barcoded particles with four open coding regions and a 7 µm microsphere attached on the bottom of the particle using COMSOL Multiphysics. Using a bottom-electrode only configuration, the barcoded particle height changes from 7 µm above the channel floor (SI Figure 3a, red) compared with the particle flowing through the middle of the channel (blue) as well as 7 µm below the channel ceiling (green). As revealed, peak strength changes with height, as particles closer to the electrodes have a greater electric field displacement. Specifically, the barcoded particle flowing closer to the bottom of the channel had a 2.27 times greater signal from coding regions and a 2.29 times greater signal from the attached 7 µm MS compared to the barcoded particle flowing in the center of the channel. Likewise, the barcoded particle flowing closer to the top of the channel had a 0.45 times lower signal from coding regions and a 0.49 times lower signal from the attached 7 µm MS compared to the barcoded particle flowing in the center of the channel. While such signal changes are important to consider, the changes that occur in electrical signals due to height variations both in particle barcoding peaks as well as the entrance and exit peaks for the particle are still identifiable no matter the particle height in the channel. It is determined that signal strength has a linear relationship with particle height in the channel, so signal peak heights can correspond with particle height in the channel and relative microsphere signal can be corrected to accurately determine their size and number of attached spheres. Assuming microfluidic flow parameters such as focused, fully-developed flow with a low Reynold's number, the barcoded particle is also likely to flow through the center of the channel with little variation over a large sample size.

As water was used for the fluid volume in every study, efforts were made to compare such results with phosphate-buffered saline (PBS), a more conventional microfluidic fluid. Here, electrical conductivity for PBS was adjusted to 0.15 S/m, compared to $5.5*10^{-6}$ S/m for water. While four orders of magnitude higher than water, the electrical conductivity for PBS is still relatively low, and simulations comparing water and PBS found no significant differences in peak electrical amplitude or peak widths (SI Figure 3b). Results using water are validated to directly translate for experimental applications using fluids like PBS.

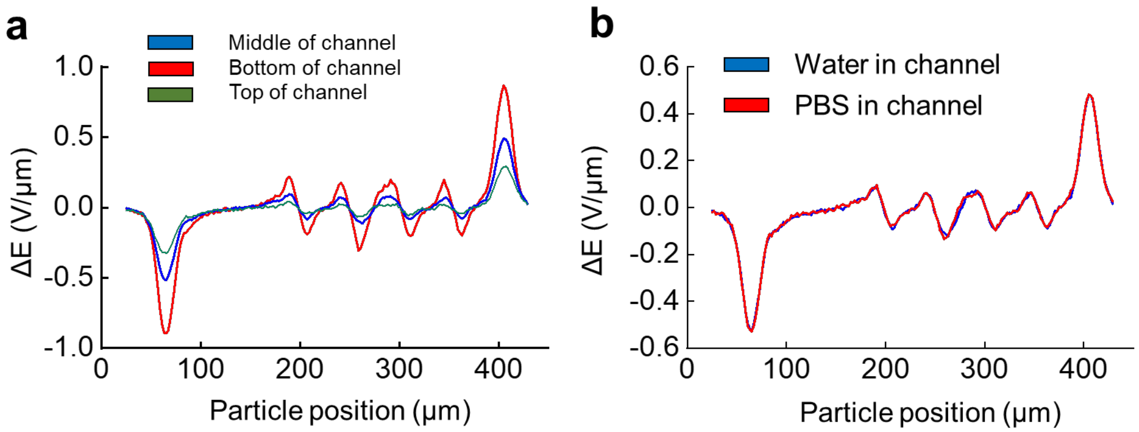

SI Figure 3: Adjusting particle height in channel and modifying volume properties. (a) Barcoded particle with 7 µm MS attached at channel center, with particle height flowing directly through the center of the channel (blue), only 7 µm from the bottom of the channel (red), and only 7 µm from the top of the channel (green) **(b)** Changing fluid media in channel as phosphate buffered saline (red) instead of water (blue) which was used for all manuscript simulations, as a barcoded particle with a 7 µm MS attached flows through the channel. As shown, peak electrical signals remain the same through changes in electrical conductivity.

References

1. Zimmerman, U., Pilwat, G., & Riemann, F. Dielectric breakdown of cell membranes. *Biophysical Journal*. **11**, 881-899 (1974).
